# Relational contexts and men’s responsibilities informing men’s participation in antenatal care in rural sub-Saharan Africa: A scoping review

**DOI:** 10.1371/journal.pgph.0005227

**Published:** 2025-09-25

**Authors:** Anthony Shuko Musiwa, Webster Mavhu, Owen Nyamwanza, Agatha Nyambi, Maya Stevens-Uninsky, Nadia Rehman, Naharin Sultana Anni, Roseline Dzekem Dine, Elizabeth Chadambuka, Rachel Couban, Lawrence Mbuagbaw

**Affiliations:** 1 Department of Health Research Methods, Evidence, and Impact, McMaster University, Hamilton, Ontario, Canada; 2 Centre for Research on Children and Familiesm, McGill University, Montreal, Quebec, Canada; 3 Department of International Public Health, Liverpool School of Tropical Medicine, Liverpool, United Kingdom; 4 Centre for Sexual Health & HIV/AIDS Research Zimbabwe, Harare, Zimbabwe; 5 Department of Global Health, McMaster University, Hamilton, Ontario, Canada; 6 Department of Community Engagement and Social Science, Rinda Ubuzima, Rwanda; 7 Department of Health Sciences, Africa University, Mutare, Zimbabwe; 8 Faculty of Health Sciences, McMaster University, Hamilton, Ontario, Canada; 9 Department of Anesthesia, McMaster University, Hamilton, Ontario, Canada; 10 Department of Pediatrics, McMaster University, Hamilton, Ontario, Canada; 11 Biostatistics Unit, Father Sean O’Sullivan Research Centre, St Joseph’s Healthcare, Hamilton, Ontario, Canada; 12 Centre for Development of Best Practices in Health, Yaoundé Central Hospital, Yaoundé, Cameroon; 13 Division of Epidemiology and Biostatistics, Department of Global Health, Stellenbosch University, Cape Town, South Africa; Worcester Polytechnic Institute, UNITED STATES OF AMERICA

## Abstract

Men’s participation is critical to improving antenatal care (ANC) and maternal and child health outcomes in sub-Saharan Africa (SSA), the region where these outcomes are the worst globally. Many current studies employ narrow, biomedical definitions that focus on men’s direct involvement in ANC. Little is known about how fatherhood and men’s participation in ANC are conceived or experienced in specific sociocultural contexts in SSA. We aimed to synthesize the existing scientific literature on the relational contexts that shape fatherhood and men’s participation in ANC, and men’s specific responsibilities within those contexts in rural SSA. Following Arksey and O’Malley’s classical methodology, we searched ten electronic databases (African Index Medicus, Africa Journals Online, CINAHL, Cochrane Library, EMBASE, MEDLINE/PubMed, PsycINFO, Sociology Collection, Social Sciences Abstract, and Social Sciences Citation Index) for peer-reviewed articles published from January 1st, 2000, to October 31st, 2024. Articles were included if they examined fatherhood and men’s participation in ANC in rural SSA, systematically analyzed primary or secondary data, and were written in any language. Seventy-seven articles reporting 58 qualitative, 6 quantitative, and 13 mixed-methods studies spanning 15 countries in SSA were included in this review. We identified ten main themes that addressed our review’s objective. Two themes depicted relational contexts that shape fatherhood experiences and men’s participation in ANC in rural SSA: (1) familial and communal collaboration, and (2) gendered and culturally-defined role structures. Eight themes described men’s specific responsibilities in ANC within the relational contexts identified above: (3) family leaders, (4) decision-makers, (5) providers, (6) protectors, (7) advocates, (8) advisors, (9) nurturers, and (10) helpers. The findings of this review highlight contextually-valid and socioculturally-meaningful experiences that broaden understandings of fatherhood and men’s participation in ANC in rural SSA. Future studies can employ Afrocentric approaches to capture often-marginalized perspectives.

## Introduction

Sub-Saharan Africa (SSA) persistently experiences the worst maternal, neonatal, and child health outcomes globally [1,2]. The region has a maternal mortality ratio of about 536 maternal deaths per 100,000 live births and an under-five mortality rate of 71 deaths per 1000 live births—both rates at least twice as high as their respective global averages [1,2]. Hence since the early 2000s, many SSA countries have implemented policies to increase the participation of men, fathers, husbands, or male spouses or partners (hereinafter, *men’s participation*) in antenatal care (ANC) and maternal and child health issues [3,4]. Men’s participation can be defined as “the involvement (…), engagement or support of men in all activities related to maternal [and child] health” [5] (p1). While evidence of its impacts on ANC utilization is mixed [6,7], men’s participation has been linked to many health benefits for mothers, children, and families, including significant reductions in risks of maternal and newborn mortality, in SSA [8–11]. Nonetheless, current reviews report that men’s participation in ANC and maternal and child health remains low across SSA [9,12]. To inform policy and practice responses, research has explored different aspects of men’s participation in ANC in SSA. These include definitions [5]; determinants [13]; facilitators, enablers, and barriers [12,14]; health and social outcomes [6–9,11]; fathers’ experiences [15,16]; and the design or impacts of interventions addressing this issue [3,10,17,18].

While insightful, many current studies employ narrow (largely Western) biomedical definitions of men’s participation that focus on men’s direct involvement, primarily male spousal accompaniment to ANC contacts, presence at birth, birth preparedness, and receipt of ANC education [5,8]. Such definitions are largely individualistic and typically exclude local cultural experiences of fatherhood and men’s participation in ANC in SSA [19,20]. In SSA, fatherhood is generally conceived as entailing responsibilities to lead, provide for, protect, and be nurturing towards women, children, and families [21,22]. These responsibilities generally differ from those expected of women and contribute to broader community efforts to raise the next generations, consistent with SSA notions of “it takes a village to raise a child” [22,23]. By neglecting these issues, studies grounded in biomedical approaches fail to account for the sociocultural relationships that shape fatherhood and men’s participation in ANC as well as men’s specific responsibilities within those relationships in SSA.

Additionally, biomedical definitions of men’s participation in ANC exclude African indigenous forms of ANC, i.e., millennia-old forms of care (e.g., traditional midwifery, traditional herbs, faith healing, spiritual rituals) provided during pregnancy drawing on African indigenous knowledge systems [24,25]. In many parts of SSA, people use location-specific African indigenous forms of ANC often concurrently with biomedical ANC [26,27]. Such practices, also known as ANC pluralism [28], reflect relational conceptions of illness, health, care, and healing and a desire for more holistic health outcomes [29,30]. During pregnancy, people interact with and receive different forms of care and support from their (extended) family and local community members, different care providers, ancestors, and other parties [24,30]. This demonstrates how pregnancy and ANC are collectively experienced in SSA [31,32]. By neglecting these pluralistic practices, studies grounded in biomedical approaches miss other important ways men participate in non-biomedical ANC contexts in SSA.

We aimed to consolidate the existing scientific literature on the relational contexts that shape fatherhood and men’s participation in ANC, and the specific responsibilities of men within those contexts in SSA. We focused on rural SSA because of its unique sociocultural dynamics compared to urban SSA [33,34]. To our knowledge, no existing literature reviews have addressed this topic. The results of this review can inform the development of policies, practice, and further research to enhance fatherhood and men’s participation in ANC in culturally-appropriate ways in rural SSA.

## Materials and methods

We employed the classical scoping review framework developed by Arksey & O’Malley [35], which has been improved over the years including by the Joanna Briggs Institute [36]. The scoping review method was the most appropriate for this study because it allows for inclusion of all relevant studies to summarize the existing knowledge about an issue [35,37]. We developed a detailed protocol, which is published elsewhere [38], to guide our review. We prepared this manuscript according to the Preferred Reporting Items for Systematic Reviews and Meta‐Analyses Extension for Scoping Reviews (PRISMA-ScR) guidelines (see S1 File) [39].

### Research questions

To guide this review, we employed the Population, Concept, Context (PCC) framework. The Population of interest was all men and women regardless of age; the Concept was fatherhood and men’s participation in ANC; and the Context was rural areas in SSA. More details of our PCC framework are outlined in Table 1 (page 3) in our published protocol [38]. Accordingly, this review addressed the following questions, which enabled us to map the range of the scientific literature relevant to the review and to identify pathways for further research:

**Table 1:** Summary of the characteristics of the included studies.

Characteristic	Number and Percentage of Studies
**Region** ^a,b^	
Eastern Africa	40 (52%)
Western Africa	26 (34%)
Northern Africa	0 (0%)
Southern Africa	14 (18%)
Central Africa	0 (0%)
**Setting**	
Community (e.g., village)	35 (45%)
Health facility (e.g., clinic)	15 (19%)
Both community and health facility	27 (35%)
**Design**	
Qualitative	58 (75%)
Quantitative	6 (8%)
Mixed methods	13 (17%)
**Approach**	
Case study	47 (61%)
Cross-sectional study	17 (22%)
Other	13 (17%)
**Data collection method** ^b^	
Focus group discussions	55 (71%)
In-depth interviews	52 (68%)
Survey questionnaire	19 (25%)

^a^ = regions based on the African Union’s grouping; ^b^ = number does not equal the total number of *included* studies since some studies included multiple countries/methods

1) How do relational contexts shape fatherhood and men’s participation in ANC in rural SSA?
2) What specific responsibilities do men have within the relational contexts identified in (1) above?

We defined “rural” as areas located outside urban centers, typically characterized by open spaces, lower population density, and a slower-paced lifestyle [40].

### Search strategy

With input from ASM and LM, RC developed the search strategy used to identify relevant studies. A detailed copy of this search strategy is attached to the published protocol as a supplemental file [38]. Using this strategy, we searched ten electronic databases: African Index Medicus, Africa Journals Online, CINAHL, Cochrane Library, EMBASE, MEDLINE/PubMed, PsycINFO, Sociology Collection, Social Sciences Abstract, and Social Sciences Citation Index. Key search terms included “men”, “fathers”, “participation”, “antenatal”, and the names of all SSA countries and regions, as well as the variations of these terms. For example, we searched MEDLINE first, using the following abbreviated strategy: (men OR fathers OR male) AND (involv* or participat* OR engage*)) AND (antenatal OR prenatal OR pregnancy OR reproductive OR family OR traditional birth attendants OR midwives OR midwifery) AND (child OR maternal) AND (sub-Saharan Africa OR names of SSA countries). We then adapted this search for the other nine databases by tailoring keywords, subject headings, and syntax to align with the indexing terms and search functions specific to each platform.

We conducted an initial search on February 28th, 2024, and a second one to update the first on October 31st, 2024. Additionally, we checked the references of all included articles to ensure our searches were thorough [41]. We imported all identified articles into an EndNote 21.4 reference library for initial screening, including deduplication, [42]. Thereafter, we moved the remaining unique articles to a DistillerSR database [43] for title/abstract and full-text screening as well as data extraction and analysis.

### Study selection

We implemented a complete dual review strategy where reviewer pairs independently screened articles to reduce bias and enhance the rigor of our findings [44,45]. All conflicts in study selection were discussed between reviewer pairs and resolved by consensus. LM acted as the tie-breaker, though no conflicts required a tie breaker. We sought only peer-reviewed articles of qualitative, quantitative, and mixed-methods studies about fatherhood and men’s participation in ANC in rural SSA published from January 1st, 2000, to October 31st, 2024. We selected articles that specifically identified the locations of their studies as rural. For those that did not specify, we inferred rurality from descriptions such as villages, remote areas, predominantly farming or pastoral areas, resettlement areas, and peri-rural settlements. We chose 2000 as the base year for the review because the emphasis in men’s participation began gaining momentum in many national and global health settings in SSA and worldwide during this time [9,13]. No language restrictions were applied. We used the PRISMA diagram to record the number of studies included and excluded at every step. This included the reasons for all exclusions to understand any biases or implications of such exclusions to our results [46].

**Fig 1:**
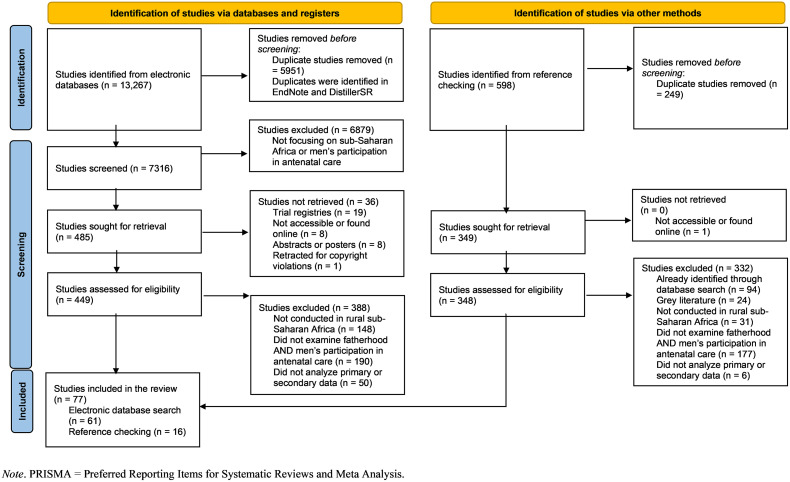
PRISMA flowchart depicting search outcomes for the scoping review.

For title/abstract screening, we started with ASM and LM separately pilot-testing our screening tool on 10 randomly-selected articles. After adjusting the tool based on this test-run, more reviewer pairs (ASM/AN, ASM/MSU, ASM/NR, ASM/NSA, ASM/ON, ASM/RDD, ASM/WM) joined to screen the remaining articles. This screening tool is presented in Section A in S2 File. Similarly, the full-text review began with ASM and LM independently piloting our screening tool on 10 studies randomly selected from those that had “passed” title/abstract screening. After revising the tool, the reviewer pairs mentioned above screened the remaining articles. A copy of this tool is outlined in Section B in S2 File. In total, our searches yielded 13,865 articles, from which 6200 duplicates were removed. After title/abstract screening, we excluded 6916 articles. After a full-text review, we excluded 720 studies, leaving 77 articles included in this review. Fig 1 provides more details about these search outcomes and reasons for exclusions.

### Data extraction

We extracted key study characteristics—namely, name(s) of author(s), year of publication, country, study setting, study objective, research approach and design, and data collection methods—as well as key findings from each study included in this review. This process began with ASM and LM test-running our data extraction tool on five articles randomly selected from the included articles. This tool was adjusted based on the piloting experience before additional reviewers (AN, MSU, NR, NSA, RDD) joined to extract data from the remaining articles. Each reviewer worked on a unique set of articles, with ASM and LM providing oversight. ASM reviewed 100% of the extracted data to ensure consistency and rigor. Any discrepancies were discussed and resolved collaboratively through consensus. A copy of our data extraction tool is presented in Section C in S2 File.

### Data analysis

We quantitatively analyzed the study characteristics extracted from the included studies using descriptive statistics such as percentages and counts. We presented this data using narratives and tables. Furthermore, using QDA Miner Lite v3.0.6 software [47], we analyzed the key findings extracted from the included studies thematically following best practices in thematic analysis, including being intentionally iterative and reflexive [48,49]. The analysis began with ASM and LM thoroughly reading and understanding the extracted data before intuitively creating and applying initial codes and themes to data extracted from 15 randomly-selected studies. Based on this initial coding and theming, the reviewers developed an initial codebook that was used to orient and guide additional reviewers (AN, MSU, NR, NSA, RDD) in coding the remaining data. Throughout, all reviewers discussed and exchanged notes that enabled them to continuously improve the thematic analysis process through, for example, creating new codes and themes or combining existing ones where there were overlaps. Finally, we integrated all our analyses, mapping out the consistencies and divergences in our data to better understand our findings and identify relevant gaps vis-à-vis our review questions [50,51]. All co-authors contributed to the development and refinement of this final analysis. We did not assess the quality of the included studies as this is not required of scoping reviews [37,52].

## Results

### Description of included studies

This review included 77 articles reporting studies conducted in 15 SSA countries and published between 2005 and 2024 (years inclusive). All articles were written in English, except one that was written in French. Slightly over half of the studies (52%) were conducted in eastern Africa while none were conducted in central and northern Africa. Tanzania (*n* = 17) had the highest number of studies, followed by Ghana (*n* = 13) and Kenya (*n* = 11). The remaining studies were distributed as follows: Uganda (*n* = 9), Nigeria (*n* = 8), Malawi (*n* = 6), Mozambique and South Africa (three each), two studies each from Ethiopia, The Gambia and Sierra Leone, and one study each from Burkina Faso, Rwanda, Zambia, and Zimbabwe. Less than half of the studies (45%) were community-based (i.e., participant recruitment and data collection were conducted outside a health facility, e.g., in a village). Three quarters of the studies (75%) were qualitative in design. At least 71% of the studies employed focus groups discussions and 68% used in-depth interviews. Table 1 summarizes the characteristics of the included studies while a more detailed description is provided in S3 File.

### Review findings

The review identified ten main themes grouped into two. Two themes depicted the relational contexts that shape fatherhood and men’s participation in ANC in rural SSA. Eight themes described men’s specific responsibilities in ANC matters within the relational contexts mentioned above. In S4 File, we provide definitions and distinctions for these themes, clarifying their conceptual boundaries and points of connection to aid interpretation and minimize overlap. In S5 File, we present the themes in table format, detailing the relational contexts (Section A in S5 File) and men’s specific responsibilities in ANC (Section B in S5 File) identified from each included article. The following sections provide a synthesis of these themes. Overall, the themes underscore contextually-valid and socioculturally-meaningful experiences of fatherhood and men’s participation in ANC in rural SSA.

### Relational contexts shaping men’s participation in ANC

#### Familial and communal collaboration.

*Shared responsibility among family members*. Twenty-three studies from Burkina Faso [53], Ethiopia [54], Ghana [31,32,55–60], Kenya [61–64], Malawi [65], Nigeria [66,67], Tanzania [68–71], and Sierra Leone [19,72] documented that ANC was often perceived as a collective responsibility among all (extended) family members, including men. Foremostly though, ANC was seen as a shared responsibility between the man and woman expecting a child, be it in marriage or out-of-wedlock settings [54,55,58,63,64,66,70]. The man and his pregnant spouse(s) were their own first line of support in navigating ANC, especially in emergency situations such as sudden illness or unexpected labor [19,31,32,59,61,67,71,73,74]. Each party had their own responsibilities but complementing each other [19,32,67]. Extended family members were seen as a second line of support [19,31,32,53,54,56,57,59–62,65,67–69,71,72]. They took part in making decisions on when or how to access what forms of ANC, or where to give birth [32,53,56,57,59,65,68,71], and helped with money for ANC or birth-related expenses, especially when the man expecting a child or their pregnant spouse had limited resources, or during emergency situations [31,53,61,65,67,71,75].

Sixteen studies in Ethiopia [54], Ghana [31,32,56,58], Kenya [62,76], Malawi [65], Nigeria [66], Sierra Leone [19,72], Tanzania [69,71,73,77], Uganda [78] revealed that senior or other experienced women from either or both the paternal and maternal extended families ([grand]mothers, mothers-in-law, sisters-in-law, aunts, co-wives, etc.) escorted pregnant women to ANC contacts or for childbirth, helped prepare for delivery, provided advice or information, or looked after or assisted the expectant women with domestic or childcare work when they were ill or could not work during the later stages of pregnancy or soon after birth. In some families or communities, expectant women went back to their parental homes during the last trimester and stayed there up to a few months after birth. During that time, they received care and support from their (grand)mothers or sisters in familiar environments [32,58,68,72]. In patrilineal societies, involving senior women from the father’s side was understood as a way of validating the paternity of and facilitating the welcoming of the new child into its family [58]. In addition to the senior women mentioned above, men looked up to other senior or experienced men in their extended families (e.g., [grand]fathers, fathers-in-law, uncles, brothers) for information, advice, or guidance around navigating pregnancy, ANC, or fatherhood [61,66,68,77].

*Collective support by community members*. Eleven studies in Ghana [58], Ethiopia [54], Malawi [74,75], Nigeria [66,67], Sierra Leone [19], and Tanzania [61,68,73,77] revealed that ANC was seen as a communal responsibility whereby local community members (e.g., friends, neighbors, workmates, churchmates) provided different kinds of support to enable families who were expecting children to navigate ANC. Community members helped pay for ANC, birthing, or transportation costs or provided actual transportation (vehicle, ox-drawn cart, etc.) to go to the ANC or birthing facility, particularly in emergency situations [67,75]. During childbirth, they gathered and celebrated together with families who had had newborns, partaking in cultural ceremonies or rituals including those for welcoming a new child [54,58]. They also shared information or their own experiences, conducted domestic or childcare work, accompanied pregnant women to ANC appointments, shared resources through income savings and lending groups or joint income generating projects, reminded expectant women about their ANC appointments or medication (e.g., for women taking HIV treatment), and provided spiritual support such as praying for each other [54,73,75,77]. Men sought information or advice or discussed their problems about fatherhood or ANC with others in their social circles [66,68,74,77]. They also leveraged their social networks to secure job opportunities or other economic resources, enabling them to meet their families’ needs during pregnancy [19].

Eleven studies conducted in Ethiopia [54], The Gambia [79], Ghana [31,32,57,59], Malawi [65], Mozambique [80], Nigeria [66], Tanzania [68], and Uganda [81] revealed that ANC providers from different healthcare systems helped men or expectant women to navigate pregnancy or ANC more effectively. Men or their expectant spouses perceived their preferred ANC providers as critical sources of advice, guidance, or information about pregnancy, ANC, childbirth, or fatherhood [31,54,66,68,80,81]. As some traditional midwives were extended family members or trusted community members with intimate knowledge of the families they served, they helped men or their pregnant women to navigate domestic conflicts or secure the resources needed to access traditional or biomedical care [32,65,81]. Three studies reported that traditional midwives acted as bridges between the biomedical and traditional healthcare systems and between these systems and families and local communities, thus facilitating access to different types of ANC [32,65,81]. They contributed to and exercised significant influence in decisions around where pregnant women gave birth and provided guidance to men on how they could support their pregnant spouses more effectively [32,59,65,79,81]. Two studies indicated that soothsayers provided families with spiritual direction around how or when to access specific forms of ANC or where to give birth [31,57].

*ANC responsibilities grounded in marriage and communal obligations*. Ten studies in Ghana [31,32,57,58], Malawi [65], Nigeria [66], Sierra Leone [19], South Africa [82], Tanzania [83], and Uganda [78] revealed that families and local communities understood collective responsibilities in ANC to be grounded in marriage and fatherhood obligations. To illustrate, one study documented that marriage gave women “demandable rights towards their husbands or even his extended kin’s support” [83] (p106). Two others reported that similar obligations applied in out-of-wedlock situations [58,82]. In addition, seven studies conducted in Ghana [31,57], Kenya [61], Nigeria [66], Sierra Leone [19], Tanzania [83], and Uganda [78] revealed that families and local communities perceived collaboration in ANC matters as grounded in the communal ways of life of African people in SSA. To illustrate, in making sense of how mothers- and sisters-in-law supported women in their families to navigate ANC, one study found that “communities in this area still live under communal settings whereby relatives live within the same area” [78] (p5). Ultimately, families and local communities, including men, collaborated in navigating ANC because they shared a sense of collective responsibility to enhance the health and wellbeing of pregnant women and children [31,32,58,65].

Nonetheless, five studies in Ghana [31,84], Malawi [75], Sierra Leone [19], South Africa [82], and Uganda [75] observed that familial or communal collaboration in ANC did not always happen due to poverty, lack of resources, divorce, cultural restrictions around marriage or out-of-wedlock relationships, or lack of willingness or preparedness to help [19,31,75,82,84]. For instance, one study in Uganda noted that the rights of married women to receive support from their spouses would be withdrawn in the event of a divorce [75]. Another in South Africa found that fathers were not allowed to perform their fathering or spousal responsibilities if they had not fulfilled certain cultural requirements such as paying *inhlawulo* (acknowledgement of paternity) when a child is born out of wedlock [82].

*Engaging with different types of ANC*. Fourteen studies in Burkina Faso [53], Ethiopia [54], The Gambia [85], Ghana [31,32,57,58,84], Kenya [86], Malawi [74,87], Nigeria [88], Sierra Leone [72], and Uganda [81] revealed that men often engaged concurrently with local African indigenous (traditional, spiritual, faith healing, etc.) and biomedical forms of ANC. However, such engagements varied based on care preferences, relationships with providers, costs, distance, and other factors. To elaborate, one study in Ghana revealed that men perceived their engagement with spiritual care practices as being much more supportive towards their pregnant spouses than escorting them to biomedical health facilities [84]. Four studies found that men preferred, encouraged, or accompanied their spouses to receive antenatal or delivery care from traditional midwives because they had good relationships with and trusted those midwives more than biomedical care providers [54,74,81,85].

Two studies documented that men took the lead or were more directly involved in consulting spiritual care than facility-based care providers [31,57]. One study in Kenya noted that men’s beliefs in traditional or faith healing reduced their likelihood of participating in biomedical prevention of mother to child transmission of HIV (PMTCT) programs [86]. Meanwhile, three studies indicated that men engaged with biomedical care for pregnancy or obstetric complications, though some consulted local traditional or spiritual care providers for convulsions which they attributed to supernatural causes like witchcraft, or due to lower costs or close proximity [53,74,88]. Finally, three studies depicted that men pinpointed health facilities as their first choice for their spouses to give birth, but some preferred their spouses to deliver at home supported by traditional midwives and other family members [31,54,72].

#### Gendered and culturally-defined role structures.

*Separate responsibilities for men and women*. Twenty-nine studies in Burkina Faso [53], Ethiopia [89], The Gambia [79], Ghana [55,59,60,90], Kenya [61,64,76,86,91], Malawi [87,92], Mozambique [80,93,94], Nigeria [66,95], Sierra Leone [19], South Africa [82], Tanzania [69,96–99], Uganda [78,100,101], and Zimbabwe [98] documented that men were seen as the leaders of or main providers for their families. They were not expected or required to accompany their pregnant spouses to care facilities but, instead, to make the necessary decisions or arrangements for their spouses to access ANC [61,67,92,95,99,102]. Besides just the information needed to facilitate access to care, men were not required to know the full details about ANC, except in emergency situations, e.g., when the mother or child was seriously ill [19, 65, 72, 80, 87, 103].

According to our review, it was considered culturally unacceptable for men to perform the roles prescribed for women (e.g., accompanying pregnant women to clinics, conducting domestic or childcare work) or to be in spaces perceived to be for women, such as ANC clinics or birthing huts [69,73,75,79,86,87,91,96,100,104,105]. Men who contravened these norms lost respect from others or were stigmatized, ridiculed, perceived as feminine or weak, or believed to be dominated or to have been bewitched by their spouses [54–58,63,64,66,69,73–76,79,80,84,86,87,90,92,94,96–100,104,106,107]. Hence, they felt uncomfortable doing women’s work or being in women’s spaces [69,73,75,79,86,87,91,96,100,104,105], though a few studies indicated that men elected to not do such work or to be in those spaces out of respect for the women [62,69,73,87].

Furthermore, 29 studies conducted in Burkina Faso [53], The Gambia [79], Ethiopia [54,89], Ghana [55,59,60,90], Kenya [61,64,76,86,91,99], Malawi [87,92], Mozambique [80,93,94], Nigeria [66], Sierra Leone [19], South Africa [82], Tanzania [69,96–98], Uganda [78,100,101], and Zimbabwe [98] observed that women were understood to be primarily responsible for domestic and childcare work. At least 40 studies reported perceptions that pregnancy or ANC were seen as female or women’s domains [19,53,55–60,63–66,69,70,72,73,75,76,78–80,82,84,86,87,91–94,96–102,104,106,108,109]. Such notions were consistent with perceptions that ANC programs and facilities were designed for and mainly targeted women [55,58–60,64,67,77,86,87,96,97,100,102,108–112]. Hence, pregnant women were seen as having the primary responsibility to go and receive ANC or birthing services, and to work with other experienced women in their (extended) families or local communities to prepare for safe delivery [19,32,56–58,63,66,69,70,73,78,82,84,86,87,93,100,102,104].

*Unequal responsibilities between men and women*. Eighteen studies from Burkina Faso [53], The Gambia [85]; Ghana [57,60,84,107,113], Kenya [64], Malawi [65,92,114], Mozambique [94]; Nigeria [67,115,116], Sierra Leone [72], and Tanzania [71,99] documented perceptions that men and women’s responsibilities in ANC were unequal and grounded in men’s control of economic resources and factors of production as well as in patriarchal or traditional norms of control, domination, force, or power over women. For example (all italics added for emphasis), one study in Sierra Leone reported that some participants “agreed that husbands have the right to *tell their wives what to do*, as he had married her and was now responsible for her” [72] (p9). Another in Kenya reported that some men felt it was acceptable to “*‘force’* their wife if she did not agree with their decisions” [64] (p7). Yet another in Mozambique noted that some men felt that escorting their pregnant spouses to ANC appointments was “necessary given their *right to control* their wives’ actions” [94] (p1725). Lastly, but not the least, one study in Nigeria commented that men’s eagerness to practice child spacing or limit parity “exemplified *patriarchal control* over the caring and managing of the women in the community” [116] (p1131).

*Shifting norms but persistent preferences for separate responsibilities*. Eighteen studies from Ethiopia [54], Ghana [31,55,58,90], Kenya [117], Malawi [87,92], Nigeria [66,95,115], Rwanda [118], Sierra Leone [19], South Africa [82], Tanzania [70,83,98,119], and Zimbabwe [98] highlighted perceptions that the gendered roles in ANC presented in the preceding sections were gradually shifting in some communities. Five studies reported a growing movement towards equal or equitable sharing of responsibilities between men and women [54,58,70,118,119]. Eleven studies depicted that some men—particularly first-time fathers, younger men, men with secondary or higher formal education levels—were more willing to perform tasks typically assigned to women, such as escorting pregnant women to clinics or conducting domestic or childcare work [19,55,58,66,87,90,92,95,98,117,118]. Four studies reported increased spousal communication, shared decision-making, and lower gender-based violence incidents during pregnancy [54,98,115,118]. One study in Ghana found that men or (expectant) women increasingly expressed reservations against the leadership or decision-making authority of compound heads or spiritual leaders around pregnancy or ANC matters [31]. The same study indicated that some women made their own decisions about ANC or childbirth [31]. Participating in formal or informal economic work outside the home enabled women to have more say in ANC matters [55,58,83,98].

Despite these shifting norms, 20 studies in Ghana [31,58,59,90,107], Kenya [63,104], Malawi [65,74,92], Mozambique [80], Nigeria [66,115], Rwanda [118], Uganda [101], South Africa [82,108], Tanzania [69,73,98], Zambia [104], and Zimbabwe [98] demonstrated that there were persistent preferences for separate roles for men and women and hesitation to embrace changing gender norms. For example, one study in Nigeria found that reports of spousal communication were not always followed by increased shared decision-making or women’s autonomy around ANC issues [115]. Another in Tanzania and Zimbabwe revealed that “more equal gender roles were perceived as a normal, although not necessarily desirable, aspect of modern living” [98] (p727). Yet another from Nigeria reported perceptions that “both men and women have culturally defined roles that remain the same, whether a woman is pregnant or not” [66] (p4). One study in South Africa revealed that, despite economic hardships, high unemployment rates, and more women working outside the home, men were still expected to provide for their families, including during pregnancy [82].

### Men’s specific responsibilities in ANC

Within the relational contexts described in the preceding sections, men were understood as having specific responsibilities in navigating ANC. Those responsibilities contributed to the collective efforts of their (expectant) spouses, extended family and local community members, and different care providers towards enhancing health and wellbeing for pregnant women, children, and families in rural SSA. The following sections present these responsibilities in more detail.

#### Family leaders.

Twenty-seven studies in Burkina Faso [53], Ethiopia [89], The Gambia [79], Ghana [55,59,60,90], Kenya [64,76,86,91], Malawi [87,92], Mozambique [80,93,94], Sierra Leone [19], Tanzania [61,69,96–99], Uganda [78,100,101], Nigeria [66], Zimbabwe [98] revealed perceptions that men led or were expected to lead their families in navigating ANC. This responsibility was said to be grounded in men’s social positions as husbands and fathers [56,57,59–61,65,70,76,77,83,85,89,107,120] or compound heads or clan elders, a role typically held by the eldest men in the whole extended family [31,57,65]. During pregnancy or childbirth, such leadership entailed the man’s duty to facilitate his pregnant spouse’s access to ANC and to ensure her overall health and wellbeing during and after pregnancy [19,55,57,58,60,61,65,68,70,72,80,121]. Echoing a previous theme, this responsibility was generally perceived to be grounded in marriage obligations [31,57,60,65,83,89], though it applied in out-of-wedlock situations too [58,82].

#### Decision-makers.

Fifteen studies from Burkina Faso [53], Ghana [57,113], Kenya [64,122], Malawi [65,74], Nigeria [67,88,115], Sierra Leone [72], and Tanzania [61,68,71,99] reported that men exercised or were expected to exercise overall authority to (dis)approve any of their spouses’ requests or decisions about care. Four studies documented that expectant women needed to obtain permission from their husbands to go to a care provider to access ANC or for delivery, or to conduct activities outside or away from the home [53,58,59,123]. One study observed that, when spouses attended ANC together, “often the man takes the lead in terms of the discussion and decisions… [and] the consultation will be directed towards the man, who having received information there, will then take care of his wife” [80] (pp7–8). Men typically undertook this responsibility with pride and acceptance that they were “responsible for the health of their families upon becoming a husband or father” [77] (p5). They derived satisfaction from knowing that “their wives are well taken care of” [66] (p4). Conversely, men felt guilty [19] and often received blame for failing to take care of their spouses or children during pregnancy [60,77,89,122].

#### Providers.

Men’s provider responsibility in ANC was by far the most salient, documented in 51 studies from Burkina Faso [53], Ethiopia [54,89], The Gambia [85], Ghana [32,55–57,59,60,84,90,107,120], Kenya [61–64,76,104,106], Malawi [65,74,75,87,92], Mozambique [80,93], Nigeria [66,67,88,95,123], Rwanda [118], Sierra Leone [19,72], South Africa [82,108], Tanzania [68–71,73,77,96,97,99,124], Uganda [75,101,121,125], and Zambia [104]. The studies depicted this responsibility as grounded in traditional cultural norms whereby fatherhood or manhood were judged primarily on men’s ability to provide for their families. In ANC, men’s responsibilities included paying the costs of ANC, birthing, and related services [19,32,53,54,56,60,62,66–69,74,77,88,89,92,95,97,99,101,104,120]. They bought the necessary medications where these had to be paid for out of pocket [19,54,56,60,62,67–69,74,77,99,101]. Men were often expected to purchase supplies needed during pregnancy and delivery, such as clothing and delivery kits [19,56,60,62,68–71,73,74,76,84,87–89,92,99,101,104,107,121]. They also provided nourishing food for pregnant women and unborn children [19,54,56,57,60–62,68–70,74,76,84,87–89,97,99,104,107]. Men often arranged or funded transportation to health facilities or African indigenous care providers for ANC, delivery, and related services [19,54,56,60–62,68,69,71–77,84,88,92,95,99,101,104,120,121]. In some cases, men paid health insurance premiums to support ANC and related costs [77]. Finally, men provided information or reminders about ANC, delivery, or related matters, such as appointments or taking medication [61,74,75,88,92,97].

#### Protectors.

Fifteen studies conducted in Ethiopia [89], Ghana [55,57], Kenya [76,86], Mozambique [80], Nigeria [66,116], Sierra Leone [19], South Africa [112], and Tanzania [61,77,83,105,126] indicated that men were or were understood as the protectors of their families. This literature noted that this responsibility was rooted in traditional cultural norms and in perceptions that men were the stronger sex and women were more vulnerable to illness or spiritual attacks during pregnancy. In carrying out this responsibility during pregnancy and ANC situations, men encouraged child spacing or parities of less than five children to protect the women from pregnancy-related complications [116]. In couples living with HIV, men practiced safer sex and advised their spouses to follow PMTCT guidelines provided by health professionals [61]. Men also assisted their spouses with domestic work to help prevent pregnancy or birth complications due to strenuous work [55,76]. Some men consulted soothsayers or conducted spiritual ceremonies to prevent illnesses or harmful spiritual attacks on the pregnant woman or unborn child [57,86]. Finally, men avoided or addressed domestic disputes in cordial ways (e.g., engaging senior family members for resolution, being patient or understanding) to prevent stress or complications [76].

Additionally, four studies demonstrated that men accompanied or arranged for other family members to accompany their pregnant spouses to ANC appointments or for birthing to protect them from harm or ensure there was someone to assist if complications arose along the way, or to ensure they received quality care once they got to the clinic [19,77,80,126]. One study in Nigeria revealed that men presented as, or were expected to be, strong for their expectant spouses by hiding their own fears or worries “in a masculine appearance of strength and courage” so that their spouses could feel assured or secure [66] (p6). Another in Ethiopia depicted that men protected (or were expected to protect) (pregnant) women and children from raids by other ethnic groups [89]. Two studies described protection from women’s perspectives, indicating that expectant women desired male accompaniment to care facilities to protect themselves from disrespectful treatment by health professionals [112,126].

#### Advocates.

Six studies from Mozambique [80], Sierra Leone [19], and Tanzania [61,69,77,126] revealed that men advocated or were expected to advocate for their spouses and families in ANC or related matters. This responsibility was closely linked to those of leadership, provision, and protection presented above. According to the review, men performed or were expected to perform this advocacy responsibility through attending ANC or delivery to ensure their spouses were treated well by care providers [69]. They requested quality care in a timely manner or spoke up against poor or unsatisfactory service delivery (e.g., long wait times, shortage of medication or supplies, disrespectful treatment) [19,61,77,80,126]. Two studies documented that men leveraged or were expected to leverage their personal and/or professional networks to facilitate access to antenatal or delivery care, or to secure opportunities (e.g., jobs) or resources to enable them to provide for their families’ ANC and related needs [19,77].

#### Advisors.

Fourteen studies conducted in Ghana [57,84], Kenya [76,104], Malawi [74], Nigeria [88,95,115], Sierra Leone [19], Uganda [81], Tanzania [61,68,73,124], and Zambia [104] demonstrated that men advised or were expected to advise their expectant spouses in navigating ANC. This responsibility was consistent with those of leadership, decision-making, provision, and protection presented above. Men performed this role out of genuine concern for the health and wellbeing of their spouses, children, and families [61,73]. In carrying out this responsibility, men shared information about which care providers (e.g., traditional midwives, spiritual healers, health professionals) to consult or how to follow care providers’ guidelines correctly [57,61,73,81]. They shared stories or experiences of other families going through similar situations to assure their spouses that they were not alone [68]. They encouraged their spouses to eat healthy foods, exercise regularly, and avoid doing heavy work [88], and reminded them about attending their ANC contacts or taking medication [61,73,74,76,88,95,104,115].

#### Nurturers.

Thirty-six studies in Ethiopia [54], Ghana [32,55–58,60,84], Kenya [61,76,104,117], Malawi [65,74,75,92], Mozambique [80,94], Nigeria [66,88,95,123], Rwanda [118], Sierra Leone [19], South Africa [82,108], Tanzania [68,77,96–98,103,124], Uganda [75,78,81], Zambia [104], and Zimbabwe [98] revealed that men were (expected to be) nurturing towards women and children. However, this responsibility was often perceived as belonging to women [19,58,76,94,98,118]. According to the reviewed literature, men were nurturing towards their expectant spouses and children because they committed to care for or support their spouses and children during the difficult times of pregnancy or childbirth [19,32,56,58,61,65,76,108]. They had a genuine concern for the health and wellbeing of their spouses and children [58,66,68,82,117]. Men perceived nurturing as an expression of care, love, emotional intimacy or support for their spouses and children during the delicate and often stressful times of pregnancy or childbirth [56,78,82,98,99,124]. They were motivated to help mitigate the added worry or stress of HIV-related stigma [61,104], share strong friendship bonds [94], and show up or be strong for, express joy towards, or make sure that their spouses were happy [56,58,66,68,78,123,124].

In carrying out this nurturing responsibility, men created peaceful, happy, or harmonious home environments; were patient and understood their spouses’ delicate, changing emotions; avoided arguments; and facilitated open or calm discussions [54,58,61,68,74,76,84,88,98,104]. They encouraged their spouses to seek care or maintain healthy habits (e.g., exercising, eating fruits), and regularly checked on them [55,74–77,81,84,88,92,95,104]. Some men accompanied their spouses to ANC contacts or for delivery [19,54,57,58,65,66,68,74,78,80,81,84,88,95,97,103,104,108,117,123,124]. Additionally, men discouraged their pregnant spouses from doing hard labor and, instead, encouraged them to rest more [19,54,55,58,61,66,68,74,84,88,97,98,108,118]. They spoiled their spouses with gifts, performed intimate acts (e.g., hugging, kissing, rubbing their bellies), or expressed companionship through spending more time with their spouses [19,61,68,98]. They also allayed their spouses’ fears and worries or assured them with affirming words and positive attitudes about the future [19,66,68,75,97,98]. In difficult situations, men often suspended or hid their own feelings, fears, or worries in order to be strong for or to make their spouses feel more secure [66,68].

#### Helpers.

Twenty-one studies from Ethiopia [54], Ghana [55,58,90], Kenya [62,76,104], Nigeria [66,88,115,123], Rwanda [118], Sierra Leone [19], South Africa [108], Tanzania [61,68,73,97,98,119], Uganda [125], Zambia [104], and Zimbabwe [98] demonstrated that, in navigating ANC, men helped or were expected to help with responsibilities traditionally assigned to women. According to this literature, men were intentionally helpful because they perceived this as their responsibility as husbands, fathers, or partners to do so [19,98,115]. They wanted to ensure all necessary work around the home was completed [98]. They were also concerned about the wellbeing of their spouses and wanted to prevent pregnancy complications due to strenuous work [55,58,76,90]. Echoing a previous theme, men wanted to create hospitable home environments or to make their spouses happy or feel good, loved, or supported throughout pregnancy [54,61,66,104]. They also chipped in because their spouses were incapacitated or had gone for ANC appointments or to give birth [58,98,125].

In carrying out this responsibility, some men performed household chores [19,54,55,58,61,62,66,68,73,76,88,90,97,98,104,108,119,125] and looked after, spent more time with, or took children for medical care when ill [55, 62, 118]. Some took up extra household economic work, e.g., spending more time on the fields or managing the portions of the farms that their spouses cultivated [19,62,68,73,76,90,97,108,115]. Others participated in group or community meetings that women typically attended, such as the *iddirs* (local support groups) in Ethiopia [54]. Others ran errands [66].

However, men’s undertaking of work typically assigned to women was often socially or culturally censored, leading some men to assist privately [66,90,98,125]. Some men did not perceive their contributions as “helping out” but, rather, as sharing responsibilities, arguing that such acts should continue outside pregnancy times [19,98,108,118]. Still, others felt that they were doing their spouses a favor because they were not obligated to engage in such work [98]. Hence, they helped out in ways that did not significantly alter gendered role structures [98].

## Discussion

Using a robust, well-validated framework [35,127], we synthesized current research on relational contexts that shape fatherhood and men’s participation in ANC, as well as men’s specific responsibilities in those contexts in rural SSA. We found that fatherhood and men’s participation in ANC occurred within and were shaped by familial and communal collaboration and gendered and culturally-defined role structures. Men were (expected or required to be) family leaders, decision-makers, providers, protectors, advocates, advisors, nurturers, and helpers. The review revealed some shifts in men’s and women’s responsibilities in ANC towards more equal or equitable gender norms, though general preferences for gendered or culturally-defined roles persisted. Some literature described men’s and women’s responsibilities in ANC as unequal gender relationships grounded in patriarchal and traditional norms of control, domination, force, or power. We discuss these findings below.

The finding that familial and communal collaboration shape experiences of ANC, fatherhood, and men’s participation in ANC in rural SSA aligns with previous research in Guatemala [128], Indonesia [129], and Iran [130]. Families, friends, neighbours, care providers (e.g., traditional midwives, religious leaders, spiritual or faith healers, biomedical health professionals) and community volunteers provide financial, material, social, moral, emotional, spiritual, and informational support to men and their expectant spouses and families to navigate ANC. This support is provided within relationships established through marriage and community, although men and their spouses are expected to be their own first line of support. It is apparent from these findings that local cultural conceptions of men’s participation in ANC across SSA reflect the prevalence of more traditional, extended family systems.

Furthermore, this review highlights the complexity and new ways of understanding fatherhood and men’s participation in ANC in rural SSA when we consider ANC pluralism. Indeed, working with their spouses, family and community members, and different care providers, men participate across African indigenous and biomedical care systems. Notions of men’s participation integrate local cultural and biomedical systems in complex ways. Such pluralism reflects an African relational and holistic model of health where pregnancy, illness, health, healing, and life in general transcend natural and spiritual worlds [28,30]. These findings echo studies that have explored the complex intersections of maternal health, non-biomedical forms of care, and men’s participation in Pakistan [131] and India [132].

The finding that gendered and culturally-defined roles shape experiences of ANC, fatherhood, and men’s participation in rural SSA is echoed in previous studies in Bangladesh [133] and Cook Island, Fiji, Papua New Guinea, Solomon Island, and Vanuatu [134]. Families and local communities in rural SSA perceive men as family leaders, decision-makers, and providers in ANC matters. They typically do not expect or require men to know much about or to be involved in the nitty-gritties of accessing ANC. Many families and communities across rural SSA see ANC as primarily women’s domains, ANC facilities as women’s spaces, and ANC as mainly targeting women. Yet, similar to studies conducted in Brazil [135], and Nepal [136], the present review indicates some shifts in these norms. For example, men—mainly younger or more formally educated men—are increasingly undertaking tasks typically reserved for women such as escorting spouses to ANC appointments or being present during birth.

Within the relational contexts discussed above, our review demonstrates that men are expected or required to perform specific responsibilities in navigating ANC in rural SSA. While we will not discuss each manifestation of these responsibilities in detail, we highlight the most salient points. Our review finds that, across rural SSA, men are understood to be the household heads or leaders of their families and (primary) decision-makers around ANC issues. They are also regarded as the main providers, with obligations to provide the resources needed for their children or expectant spouses to access care and stay healthy. These findings are reflected in studies conducted in Iran [130] and Pakistan [137]. In addition, similar to findings in previous research in Bangladesh [138] and India [139], some men in rural SSA escort their expectant spouses to ANC facilities and share emotional intimacy, care, and love with them, including by openly discussing their spouses’ concerns. Such aspects reflect men’s responsibilities to protect and nurture their spouses and families. Our review also indicates that men in rural SSA provide critical advice and information. They assist their pregnant spouses with domestic and childcare work to allow them to rest more and to prevent negative pregnancy outcomes, similar to previous research in Nepal [136] and Papua New Guinea [140].

Nonetheless, some studies in our review find that the responsibilities of men and women in ANC are seen as characterized by gender inequalities rooted in patriarchal or traditional cultural norms of control, domination, force, or power over women. Previous studies in Bangladesh [133], Pakistan [137], and Jordan and Saudi Arabia [141] documented similar understandings. While our review finds some shifts in these responsibilities towards more equal or equitable role sharing, there remains a general preference for gendered role distributions in many rural parts of SSA. A study in Pakistan reported similar notions, stating that while gender roles and relationships were negotiable and varied between contexts, Pakistani society remained largely stratified along gender and class lines [137].

Ultimately, our review sheds at least three critical insights that build on existing knowledge. These insights indicate opportunities to center local ways of fostering fatherhood and men’s ANC participation in rural SSA. First, fatherhood and men’s participation in ANC in rural SSA cannot be understood fully without considering the familial and communal settings of African ways of being and living [23,142]. Men have always had specific responsibilities towards the health and wellbeing of women, children, and families. In ANC contexts, they contribute by performing specific culturally-defined responsibilities such as being providers and protectors. Yet, biomedical definitions of men’s participation often neglect those responsibilities. Such definitions reflect individualistic and gender-neutral conceptions whereby men are expected to be directly involved in *biomedical* ANC activities.

Second, understanding fatherhood and men’s participation in ANC in rural SSA requires paying attention to the pluralistic ways families experience pregnancy, childbirth, illness, health, healthcare, or healing. As families often mix traditional, spiritual, faith-based, and biomedical forms of care in pregnancy or childbirth [26,28], understandings of fatherhood and men’s participation must be broadened beyond biomedical contexts. Finally, despite perceptions that fatherhood and men’s participation in ANC in rural SSA is grounded in control, domination, force, or power over women, our review finds that men enact their responsibilities with good intentions to ensure the health and wellbeing of women, children, and families. This latter finding suggests a rarely acknowledged outlook towards this issue, one that reflects *Ubuntu*, a way of living and being that promotes equal rights, equitable gender norms, health-promoting behaviors, and human flourishing based on African cultural values [143–145].

### Implications for policy, practice, and further research

We recommend that policymakers and practitioners engage in genuine dialogue and collaboration with men, women, families, and local communities to reimagine and promote men’s participation in ANC in ways that reflect local lived realities in rural areas in SSA. While there may be some tensions, different ANC systems and approaches present important opportunities to leverage their unique strengths to enhance men’s participation in ANC across rural SSA.

Policymakers and practitioners should meet families where they are in their efforts to enhance men’s participation in ANC in rural SSA [146,147]. Policies and practice should employ family or communal systems approaches [148,149] in supporting men (and women) to more effectively perform their expected responsibilities in ANC within their familial or communal networks. For example, such efforts should strengthen men’s responsibilities to provide for their families during pregnancy through enhancing local subsistence economies (e.g., farming, mining) and employment opportunities. At the same time, policy and practice interventions should strengthen the support provided by other parties to complement men’s contributions in ANC matters in rural SSA. For instance, a few studies in this review [31,32,58,69] and elsewhere outside SSA [150,151] indicate that (grand)mothers and in-laws play critical social, emotional, and spiritual support roles in maternal health matters.

Policymakers and practitioners should provide community and formal education opportunities in more respectful and culturally sensitive ways to encourage men (and women) to embrace the kinds of participation defined in biomedical guidelines. A few studies in this review (e.g., [66,90,92]) suggest that men and women who are exposed to biomedical ANC information or have higher formal education levels tend to perceive male spousal accompaniment to clinics or men undertaking roles traditionally assigned to women more positively. The education programs recommended here could draw lessons from the positive impacts of behavior change strategies (education, incentivization, modelling, etc.) in maternal and child health [152]. To be more impactful, they should be gender-transformative [17,18,143,144] and grounded in *Ubuntu* [143,144], targeting all family and community members.

Finally, we underline some critical research directions. More research around fatherhood and men’s participation in ANC is needed generally in SSA and more so in central and southern Africa. Importantly, most studies included in this review only tangentially touched (e.g., as one of several themes or as a discussion point) on how experiences or conceptions of fatherhood shape men’s participation in ANC in specific rural areas in SSA. More in-depth research on these issues is needed. Cuing from a few studies in this review that explored this terrain, future research can use qualitative designs to examine how the complex interactions between fatherhood, masculinities, and structural factors shape men’s participation in ANC [82], how gender dynamics play out when families are expecting a baby [85,90], how men and women conceptualize participation in ANC in their specific localities [19,76,108], and how community, familial, social, and traditional structures shape men’s participation in ANC [31,32,79].

We noted that studies that examined fatherhood experiences and local socio-cultural contexts in greater depth were able to illuminate men’s participation in local African indigenous forms of ANC such as traditional midwifery, faith healing, and spiritual care (e.g., [31,32,57,81]). Insights from these studies reflect the ANC pluralism that exists in many (rural) parts of SSA [29,153]. Future research can employ postcolonial Afrocentric [143,144] and Afro-feminist [154] approaches that center these African realities and highlight the often-neglected ways men participate and collaborate with other parties in ANC in rural SSA. Qualitative research that provides more in-depth insights and quantitative studies that estimate rates of men’s participation in non-biomedical ANC settings would be insightful.

### Limitations

This review has some limitations. We reviewed peer-reviewed articles only. While we may have missed other important insights by excluding other forms of literature including grey literature, we believe that such exclusion did not significantly alter our findings. This is because our search strategies yielded a large number of peer-reviewed articles which saturated our data. Another limitation is that studies included in this review were from different cultural and geographical contexts in rural SSA and employed different methods. Our analysis synthesized the findings from these studies and, in doing so, lost the specific nuances of fatherhood and men’s ANC participation in specific rural contexts in SSA. Some of our suggested research directions could help address those missed nuances.

## Conclusion

In this scoping review, we aimed to synthesize current research around fatherhood and men’s participation in ANC in rural SSA. We found that these issues occurred within and were shaped by relational contexts characterized by familial and communal collaboration as well as gendered and culturally-defined role structures. Within those contexts, men were (expected to be) family leaders, decision-makers, providers, protectors, advocates, advisors, nurturers, and helpers. There were some shifts or overlaps between men’s and women’s ANC responsibilities toward more equal or equitable sharing of roles, but preferences for culturally-defined or gendered role structures persisted. There were also perceptions that fatherhood and men’s responsibilities were grounded in patriarchal or traditional norms of control, domination, force, or power over women. Yet, it appears that men performed their responsibilities with good intentions. Ultimately, this review underscores contextually valid and culturally meaningful conceptions and experiences that broaden our understanding of fatherhood and men’s participation in ANC in rural SSA. Policies, practice, and research that center these conceptions and experiences as strengths rather than deficits can yield more significant impacts on fatherhood, men’s participation, and maternal and child health in SSA.

## Supporting information

S1 File Preferred Reporting Items for Systematic reviews and Meta-Analyses extension for Scoping Reviews (PRISMA-ScR) Checklist. (DOCX)

S2 File Screening and data extraction tools. (DOC)

S3 File Characteristics of included studies. (DOC)

S4 File Thematic map of relational contexts and perceived responsibilities informing men’s participation in antenatal care in rural sub-Saharan Africa. (DOCX)

S5 File Findings from the included studies. (DOCX)
