# Supplementary material for: Relational contexts and men’s responsibilities informing men’s participation in antenatal care in rural sub-Saharan Africa: A scoping review
Source: PLOS Glob Public Health. 2025 Sep 25;5(9):e0005227. doi: 10.1371/journal.pgph.0005227 (PMC12463206; doi:10.1371/journal.pgph.0005227)
Supplement: S4 File — (DOCX) [file pgph.0005227.s004.docx]

# **S4 File. Thematic map of relational contexts and perceived responsibilities informing men’s participation in antenatal care in rural sub-Saharan Africa.**

| **No.** | **Theme** | **Definition** | **Distinctive Focus** | **Related but Distinct Themes** |
| --- | --- | --- | --- | --- |
| ***Relational contexts shaping men’s participation in ANC*** | | | | |
| **1** | Familial and Communal Collaboration | Pregnancy and ANC are understood as collective responsibilities shared among family and community members. | Highlights the collective and relational structure of ANC involvement. | Gendered and Culturally-Defined Role Structures |
| **2** | Gendered and Culturally-Defined Role Structures | Roles in ANC are influenced by traditional norms assigning different responsibilities to men and women. | Emphasizes how cultural norms shape expectations and limitations of participation. | Familial and Communal Collaboration |
| ***Men’s specific responsibilities in ANC*** | | | | |
| **3** | Family Leaders | Men are viewed as heads of families and are expected to lead ANC decision-making and processes. | Focuses on hierarchical family leadership by men. | Decision-Makers, Providers |
| **4** | Decision-Makers | Men are responsible for authorizing or denying ANC-related actions. | Authority in approving or guiding ANC-related decisions. | Family Leaders, Advisors |
| **5** | Providers | Men are expected to provide financial and material support for ANC and childbirth. | Economic responsibility to facilitate care access. | Protectors, Helpers |
| **6** | Protectors | Men ensure safety and wellbeing of pregnant women, including spiritual or physical protection. | Safeguarding against harm, ensuring care environment is secure. | Providers, Advocates |
| **7** | Advocates | Men speak on behalf of spouses/families to care providers and institutions. | Externally-facing support, navigating institutions. | Advisors, Protectors |
| **8** | Advisors | Men offer guidance, reminders, and information about ANC to spouses. | Internal family-level guidance and mentoring. | Advocates, Decision-Makers |
| **9** | Nurturers | Men provide emotional support and contribute to household harmony during pregnancy. | Emotional intimacy, care, and well-being. | Helpers, Advisors |
| **10** | Helpers | Men engage in domestic and childcare work to support pregnant spouses. | Practical, hands-on contributions to household tasks. | Nurturers, Providers |

*Notes*: ANC = antenatal care; SSA = sub-Saharan Africa
