# Supplementary material for: Relational contexts and men’s responsibilities informing men’s participation in antenatal care in rural sub-Saharan Africa: A scoping review
Source: PLOS Glob Public Health. 2025 Sep 25;5(9):e0005227. doi: 10.1371/journal.pgph.0005227 (PMC12463206; doi:10.1371/journal.pgph.0005227)
Supplement: S5 File — (DOCX) [file pgph.0005227.s005.docx]

# **S5 File. Findings from the included studies.**

## **Section A. Themes describing the relational contexts that shape men’s participation in ANC identified in each included study.**

| **Main theme** | **Familial and communal collaboration** | | | | **Gendered and culturally-defined role structures** | | |
| --- | --- | --- | --- | --- | --- | --- | --- |
| **Sub-theme** | Shared responsibility among family members | Collective support by community members | ANC responsibilities grounded in marriage and communal obligations | Engaging with different types of ANC | Separate responsibilities for men and women | Unequal responsibilities between men and women | Shifting norms but persistent preferences for separate responsibilities. |
| **Mullick et al., 2005 [111]** |  |  |  |  | **✓** |  |  |
| **Odimegwu et al., 2005 [87]** |  |  |  | **✓** |  |  |  |
| **Jansen, 2006 [32]** | **✓** | **✓** |  | **✓** | **✓** |  |  |
| **Mbweza et al., 2008 [113]** |  |  |  |  |  | **✓** |  |
| **Pembe et al., 2008 [70]** | **✓** |  |  |  |  | **✓** |  |
| **Olayemi et al., 2009 [94]** |  |  |  |  | **✓** |  | **✓** |
| **Theuring et al., 2009 [104]** |  |  |  |  | **✓** |  |  |
| **Byamugisha et al., 2010 [108]** |  |  |  |  | **✓** |  |  |
| **Reece et al., 2010 [85]** |  |  |  | **✓** | **✓** |  |  |
| **Adeleye et al., 2011 [115]** |  |  |  |  |  | **✓** |  |
| **Falnes et al., 2011 [99]** |  |  |  |  | **✓** |  |  |
| **Abass et al., 2012 [106]** |  |  |  |  | **✓** | **✓** | **✓** |
| **Kululanga et al., 2012 [86]** |  |  |  | **✓** | **✓** |  | **✓** |
| **Aarnio et al., 2013 [73]** |  | **✓** |  | **✓** | **✓** |  | **✓** |
| **Gross et al., 2013 [82]** |  |  | **✓** |  |  |  | **✓** |
| **Kwambai et al., 2013 [62]** | **✓** |  |  |  | **✓** | **✓** |  |
| **Somé et al., 2013 [52]** | **✓** |  |  | **✓** | **✓** | **✓** |  |
| **Doyle et al., 2014 [117]** |  |  |  |  |  |  | **✓** |
| **Dumbaugh et al., 2014 [59]** | **✓** |  |  |  | **✓** | **✓** |  |
| **McMahon et al., 2014 [125]** |  |  |  |  |  |  |  |
| **Moyer et al., 2014 [31]** | **✓** | **✓** | **✓** | **✓** |  |  | **✓** |
| **Singh et al., 2014 [100]** |  |  |  |  | **✓** |  | **✓** |
| **Azuh et al., 2015 [66]** | **✓** | **✓** |  |  | **✓** | **✓** |  |
| **Ganle & Dery, 2015 [58]** | **✓** | **✓** |  |  | **✓** |  | **✓** |
| **Ganle et al., 2015 [112]** |  |  |  |  |  | **✓** |  |
| **Audet et al., 2016 [93]** |  |  |  |  | **✓** | **✓** |  |
| **Brubaker et al., 2016 [75]** | **✓** |  |  |  | **✓** |  |  |
| **Ganle et al., 2016 [119]** |  |  |  |  |  |  |  |
| **Lowe et al., 2016 [84]** |  |  |  | **✓** |  | **✓** |  |
| **Nyandieka et al., 2016 [121]** |  |  |  |  |  |  |  |
| **Turinawe et al., 2016 [80]** |  | **✓** |  | **✓** |  |  |  |
| **Vermeulen et al., 2016 [69]** | **✓** |  |  |  | **✓** |  | **✓** |
| **Bougangue & Ling, 2017 [57]** | **✓** | **✓** |  | **✓** | **✓** |  | **✓** |
| **Flax et al., 2017 [74]** |  | **✓** | **✓** |  | **✓** |  |  |
| **Lowe, 2017 [78]** |  | **✓** |  |  | **✓** |  |  |
| **Manda-Taylor et al., 2017 [91]** |  |  |  |  | **✓** | **✓** | **✓** |
| **Matseke et al., 2017 [107]** |  |  |  |  | **✓** |  | **✓** |
| **Morgan et al., 2017 [120]** |  |  |  |  |  |  |  |
| **Sileo et al., 2017 [110]** |  |  |  |  | **✓** |  |  |
| **Aarnio et al., 2018 [64]** | **✓** | **✓** |  |  | **✓** | **✓** | **✓** |
| **Aborigo et al., 2018 [56]** | **✓** | **✓** | **✓** | **✓** | **✓** | **✓** |  |
| **Maluka & Peneza, 2018 [68]** | **✓** |  |  |  | **✓** |  | **✓** |
| **Musoke et al., 2018 [62]** | **✓** |  |  |  | **✓** |  | **✓** |
| **Treacy et al., 2018 [71]** | **✓** |  |  | **✓** | **✓** | **✓** |  |
| **Wombeogo & Ayembilla, 2018 [83]** |  |  | **✓** | **✓** | **✓** | **✓** |  |
| **Cheptum et al., 2019 [61]** | **✓** |  |  |  | **✓** |  |  |
| **Galle et al., 2019 [77]** |  | **✓** |  |  | **✓** |  | **✓** |
| **Greenspan et al., 2019 [76]** | **✓** | **✓** |  |  | **✓** |  |  |
| **Kayongo & Miller, 2019 [124]** |  |  |  |  |  |  |  |
| **Muheirwe & Nuhu, 2019a [99]** |  |  |  |  | **✓** |  |  |
| **Muheirwe & Nuhu, 2019b [75]** | **✓** |  | **✓** |  | **✓** |  |  |
| **Ongolly & Bukachi, 2019 [90]** |  |  |  |  | **✓** |  |  |
| **Saah et al., 2019 [55]** | **✓** |  |  |  | **✓** |  |  |
| **Al-Mujtaba et al., 2020 [122]** |  |  |  |  |  |  |  |
| **Comrie-Thomson et al., 2020 [97]** |  |  |  |  | **✓** |  | **✓** |
| **Gibore & Bali, 2020 [72]** | **✓** | **✓** |  |  | **✓** |  | **✓** |
| **Mabachi et al., 2020 [60]** | **✓** | **✓** | **✓** |  | **✓** |  |  |
| **Maluka, Japhet, et al., 2020 [123]** |  |  |  |  |  |  |  |
| **Maluka, Joseph, et al., 2020 [98]** |  |  |  |  | **✓** | **✓** |  |
| **McLean, 2020 [19]** | **✓** | **✓** | **✓** |  | **✓** |  | **✓** |
| **Onyeze-Joe & Godin, 2020 [65]** | **✓** | **✓** | **✓** |  | **✓** |  | **✓** |
| **Tinuola et al., 2020 [109]** |  |  |  |  | **✓** |  |  |
| **Boniphace et al., 2021 [102]** |  |  |  |  | **✓** |  |  |
| **Chahalis et al., 2021 [118]** |  |  |  |  |  |  | **✓** |
| **Gibore & Gesase, 2021 [96]** |  |  |  |  | **✓** |  |  |
| **Jeong et al., 2021 [92]** |  |  |  |  | **✓** |  |  |
| **Lusambili, Muriuki, et al., 2021 [105]** |  |  |  |  | **✓** |  |  |
| **Lusambili, Wisofschi, et al., 2021 [116]** |  |  |  |  |  |  | **✓** |
| **Mudi et al., 2021 [114]** |  |  |  |  |  | **✓** | **✓** |
| **Ampim et al., 2022 [89]** |  |  |  |  | **✓** |  | **✓** |
| **Kaba et al., 2022 [88]** |  |  |  |  | **✓** |  |  |
| **Boniphace et al., 2022 [95]** |  |  |  |  | **✓** |  |  |
| **Mooij et al., 2022 [67]** | **✓** | **✓** |  |  |  |  |  |
| **Morgan et al., 2022 [54]** | **✓** |  |  |  | **✓** |  | **✓** |
| **Hampanda et al., 2024 [103]** |  |  |  |  | **✓** |  | **✓** |
| **Makusha, 2024 [81]** |  |  | **✓** |  | **✓** |  | **✓** |
| **Yarinbab et al., 2024 [53]** | **✓** | **✓** |  | **✓** | **✓** |  | **✓** |

*Note*: * = the number in brackets in front of each article corresponds to its number in the references list; ANC = antenatal care

## **Section B. Themes describing men’s specific responsibilities in ANC identified in each included study.**

| **Main theme** | **Family leaders** | **Decision makers** | **Providers** | **Protectors** | **Advocates** | **Advisors** | **Nurturers** | **Helpers** |
| --- | --- | --- | --- | --- | --- | --- | --- | --- |
| **Mullick et al., 2005 [111]** |  |  |  | **✓** |  |  |  |  |
| **Odimegwu et al., 2005 [87]** |  | **✓** | **✓** |  |  | **✓** | **✓** | **✓** |
| **Jansen, 2006 [32]** |  |  | **✓** |  |  |  | **✓** |  |
| **Mbweza et al., 2008 [113]** |  |  |  |  |  |  |  |  |
| **Pembe et al., 2008 [70]** |  | **✓** | **✓** |  |  |  |  |  |
| **Olayemi et al., 2009 [94]** |  |  | **✓** |  |  | **✓** | **✓** |  |
| **Theuring et al., 2009 [104]** |  |  |  | **✓** |  |  |  |  |
| **Byamugisha et al., 2010 [108]** |  |  |  |  |  |  |  |  |
| **Reece et al., 2010 [85]** | **✓** |  |  | **✓** |  |  |  |  |
| **Adeleye et al., 2011 [115]** |  |  |  | **✓** |  |  |  |  |
| **Falnes et al., 2011 [101]** |  |  |  |  |  |  |  |  |
| **Abass et al., 2012 [106]** | **✓** |  | **✓** |  |  |  |  |  |
| **Kululanga et al., 2012 [86]** | **✓** |  | **✓** |  |  |  |  |  |
| **Aarnio et al., 2013 [73]** |  | **✓** | **✓** |  |  | **✓** | **✓** |  |
| **Gross et al., 2013 [82]** | **✓** |  |  | **✓** |  |  |  |  |
| **Kwambai et al., 2013 [63]** | **✓** | **✓** | **✓** |  |  |  |  |  |
| **Somé et al., 2013 [52]** | **✓** | **✓** | **✓** |  |  |  |  |  |
| **Doyle et al., 2014 [117]** |  |  | **✓** |  |  |  | **✓** | **✓** |
| **Dumbaugh et al., 2014 [59]** | **✓** | **✓** | **✓** |  |  |  | **✓** |  |
| **McMahon et al., 2014 [125]** |  |  |  | **✓** | **✓** |  |  |  |
| **Moyer et al., 2014 [31]** | **✓** |  |  |  |  |  |  |  |
| **Singh et al., 2014 [100]** | **✓** |  | **✓** |  |  |  |  |  |
| **Azuh et al., 2015 [66]** |  | **✓** | **✓** |  |  |  |  |  |
| **Ganle & Dery, 2015 [58]** | **✓** | **✓** | **✓** |  |  |  |  |  |
| **Ganle et al., 2015 [112]** |  | **✓** |  |  |  |  |  |  |
| **Audet et al., 2016 [93]** | **✓** |  |  |  |  |  | **✓** |  |
| **Brubaker et al., 2016 [75]** | **✓** |  | **✓** | **✓** |  | **✓** | **✓** | **✓** |
| **Ganle et al., 2016 [119]** | **✓** |  | **✓** |  |  |  |  |  |
| **Lowe et al., 2016 [84]** | **✓** |  | **✓** |  |  |  |  |  |
| **Nyandieka et al., 2016 [121]** |  | **✓** |  |  |  |  |  |  |
| **Turinawe et al., 2016 [80]** |  |  |  |  |  | **✓** | **✓** |  |
| **Vermeulen et al., 2016 [69]** | **✓** |  | **✓** |  |  |  |  |  |
| **Bougangue & Ling, 2017 [57]** | **✓** | **✓** |  |  |  |  | **✓** | **✓** |
| **Flax et al., 2017 [74]** |  |  | **✓** |  |  |  | **✓** |  |
| **Lowe, 2017 [78]** | **✓** |  |  |  |  |  |  |  |
| **Manda-Taylor et al., 2017 [91]** | **✓** |  | **✓** |  |  |  | **✓** |  |
| **Matseke et al., 2017 [107]** |  |  | **✓** |  |  |  | **✓** | **✓** |
| **Morgan et al., 2017 [120]** | **✓** |  | **✓** |  |  |  |  |  |
| **Sileo et al., 2017 [110]** |  |  |  |  |  |  |  |  |
| **Aarnio et al., 2018 [64]** | **✓** | **✓** | **✓** |  |  |  | **✓** |  |
| **Aborigo et al., 2018 [56]** | **✓** | **✓** | **✓** | **✓** |  | **✓** | **✓** |  |
| **Maluka & Peneza, 2018 [68]** | **✓** |  | **✓** |  | **✓** |  |  |  |
| **Musoke et al., 2018 [62]** |  |  | **✓** |  |  |  |  |  |
| **Treacy et al., 2018 [71]** | **✓** | **✓** | **✓** |  |  |  |  |  |
| **Wombeogo & Ayembilla, 2018 [83]** |  |  | **✓** |  |  | **✓** | **✓** |  |
| **Cheptum et al., 2019 [61]** |  |  | **✓** |  |  |  |  | **✓** |
| **Galle et al., 2019 [79]** | **✓** | **✓** | **✓** | **✓** | **✓** |  | **✓** |  |
| **Greenspan et al., 2019 [72]** | **✓** | **✓** | **✓** | **✓** | **✓** |  | **✓** |  |
| **Kayongo & Miller, 2019 [124]** |  |  | **✓** |  |  |  |  | **✓** |
| **Muheirwe & Nuhu, 2019a [99]** | **✓** |  |  |  |  |  |  |  |
| **Muheirwe & Nuhu, 2019b [77]** | **✓** |  |  |  |  |  | **✓** |  |
| **Ongolly & Bukachi, 2019 [90]** | **✓** |  |  |  |  |  |  |  |
| **Saah et al., 2019 [55]** | **✓** |  | **✓** |  |  |  | **✓** |  |
| **Al-Mujtaba et al., 2020 [122]** |  | **✓** | **✓** |  |  |  | **✓** | **✓** |
| **Comrie-Thomson et al., 2020 [97]** | **✓** |  |  |  |  |  | **✓** | **✓** |
| **Gibore & Bali, 2020 [72]** |  |  | **✓** |  |  | **✓** |  | **✓** |
| **Mabachi et al., 2020 [60]** | **✓** | **✓** | **✓** | **✓** | **✓** | **✓** | **✓** | **✓** |
| **Maluka, Japhet, et al., 2020 [123]** |  |  | **✓** |  |  | **✓** | **✓** |  |
| **Maluka, Joseph, et al., 2020 [98]** | **✓** | **✓** | **✓** |  |  |  |  |  |
| **McLean, 2020 [19]** | **✓** |  | **✓** | **✓** | **✓** | **✓** | **✓** | **✓** |
| **Onyeze-Joe & Godin, 2020 [65]** | **✓** | **✓** | **✓** | **✓** |  |  | **✓** | **✓** |
| **Tinuola et al., 2020 [109]** |  |  |  |  |  |  |  |  |
| **Boniphace et al., 2021 [102]** |  |  |  |  |  |  | **✓** |  |
| **Chahalis et al., 2021 [118]** |  |  |  |  |  |  |  | **✓** |
| **Gibore & Gesase, 2021 [96]** | **✓** |  | **✓** |  |  |  | **✓** | **✓** |
| **Jeong et al., 2021 [92]** | **✓** |  | **✓** |  |  |  |  |  |
| **Lusambili, Muriuki, et al., 2021 [105]** |  |  | **✓** |  |  |  |  |  |
| **Lusambili, Wisofschi, et al., 2021 [116]** |  |  |  |  |  |  | **✓** |  |
| **Mudi et al., 2021 [114]** |  | **✓** |  |  |  | **✓** |  | **✓** |
| **Ampim et al., 2022 [89]** | **✓** |  | **✓** |  |  |  |  | X |
| **Kaba et al., 2022 [88]** | **✓** | **✓** | **✓** | **✓** |  |  |  |  |
| **Boniphace et al., 2022 [95]** | **✓** |  | **✓** |  |  |  | **✓** |  |
| **Mooij et al., 2022 [67]** | **✓** | **✓** | **✓** |  |  | **✓** | **✓** | **✓** |
| **Morgan et al., 2022 [54]** | **✓** |  | **✓** | **✓** |  |  | **✓** | **✓** |
| **Hampanda et al., 2024 [103]** |  |  | **✓** |  |  | **✓** | **✓** | **✓** |
| **Makusha, 2024 [81]** | **✓** |  | **✓** |  |  |  | **✓** |  |
| **Yarinbab et al., 2024 [53]** |  |  | **✓** |  |  |  | **✓** | **✓** |

*Note*: * = the number in brackets in front of each article corresponds to its number in the references list; ANC = antenatal care
